# Qualitative research: the heart of implementation studies

**DOI:** 10.1590/0034-7167.20267902

**Published:** 2026-02-23

**Authors:** Ellen Synthia Fernandes de Oliveira, Maria Helena Presado, Cristina Lavareda Baixinho

**Affiliations:** I Professor in the Graduate Program in Public Health at Universidade Federal de Goiás – Brazil. Post-Doctoral Degree in Public Health from the School of Medical Sciences at Universidade Estadual de Campinas – Brazil.; II Nursing Professor at Escola Superior de Enfermagem de Lisboa – Portugal. Researcher at Centro de Investigação, Inovação e Desenvolvimento em Enfermagem de Lisboa, Lisbon, Portugal.; III Nursing Professor at Escola Superior de Enfermagem de Lisboa – Portugal. Doctoral Degree in Nursing. Researcher at Centro de Investigação, Inovação e Desenvolvimento em Enfermagem de Lisboa, Lisbon, and at Centro de Inovação em Tecnologias e Cuidados de Saúde, Leiria, Portugal.

## INTRODUCTION

In general, implementation studies in health, and in nursing in particular, face challenges that reflect the complexity of clinical and organizational contexts, as well as the interaction among the different actors involved in knowledge production, dissemination, and application^(1)^. The complexity of healthcare systems and interpersonal and institutional relationships, recognized by the World Health Organization itself^(2)^, prevents health decision-making from being consistently informed by the best available evidence, while simultaneously considering the preferences and values of individuals and their families, as well as social perception, fairness, feasibility of implementation, equitable access, sustainability and acceptance by those involved^(1–3)^.

The solution to this complex problem lies in the effective involvement of healthcare recipients in all phases of the knowledge translation process, from planning and developing projects to implementing and maintaining knowledge use, aiming to generate societal impact^(1–3)^. Thus, research should not only produce new results, but also promote a deep understanding of the needs of contexts and people, identifying barriers and facilitators to knowledge transfer^(3)^.

In light of the above, qualitative research proves fundamental to understanding the perceptions, motivations, and barriers experienced by all actors involved throughout the different phases of the knowledge translation process^(1)^. This approach allows strategies to be adapted to the specific realities of healthcare services, promoting a more reflective, contextualized, and participatory practice.

In highly complex contexts, implementation studies that incorporate a qualitative approach play an essential role in understanding how planned interventions—whether public policies, health programs, or educational practices—materialize in practice, also allowing for the assessment of their effectiveness. Contrary to the prevailing positivist view, according to which effectiveness is measured solely by numerical indicators, qualitative research seeks to understand the “why” and “how” of human actions and experiences. Thus, this approach becomes indispensable, as it illuminates meanings, perceptions and human relationships that underpin any change process^(1,3)^, contributing to health practice being guided by up-to-date and contextualized knowledge.

This perspective broadens the understanding of how evidence is transformed into action and sustained in different care contexts. In implementation studies, this qualitative approach makes it possible to understand why a well-designed intervention does not produce the expected impact or how a seemingly simple practice is reinterpreted or appropriated by professionals and communities. In this way, qualitative research offers a mirror of the social, cultural, and organizational dynamics that shape the reality of implementation^(3)^.

The adoption of naturalistic, interpretative, and holistic approaches is therefore imperative. These approaches promote in-depth analysis, enrich knowledge and methodological innovation, and support ethical and interdisciplinary decision-making. These characteristics have likely contributed to the growing prominence of mixed methods studies, which seek to integrate the best of both paradigms—quantitative and qualitative—into a more comprehensive and complementary vision.

In this context, the qualitative approach takes over a central role, not only for its ability to deepen the understanding of phenomena, but also for its potential to transform practices and relationships in healthcare. Qualitative research also has a transformative effect on implementation studies, as it gives voice and space to different participants—those being cared for and their families, as well as professionals, managers, and policymakers—promoting a more democratic, inclusive, and participatory science. More than just producing knowledge about implementation, qualitative research contributes to improving the processes themselves by allowing stakeholders to critically reflect on their practices. This reflective dimension transforms research into a space for learning and meaning-making.

In short, designating qualitative research as the “soul” of implementation studies is not merely a poetic metaphor, but a recognition of its essential role in understanding the human dimensions of change. Policies and programs only come to life when they are interpreted, adapted, and lived by people. It is attentive listening, a sensitive gaze, and in-depth analysis that reveal what numbers do not show: the meanings that underpin actions. Thus, qualitative research is not just a method, but a way of seeing and understanding the world—a science of the human that illuminates the true soul of implementation.
