# Supplementary material for: Qualitative research: the heart of implementation studies
Source: Rev Bras Enferm. 2026 Feb 23;79:e7902. doi: 10.1590/0034-7167.20267902 (PMC12933931; doi:10.1590/0034-7167.20267902)
Supplement: Translation

# Translation: Investigação qualitativa: a alma dos estudos de implementação

**DOI:** 10.1590/0034-7167.20267902pt

**Authors:** Ellen Synthia Fernandes de Oliveira, Maria Helena Presado, Cristina Lavareda Baixinho

**Affiliations:** I Docente do Programa de Pós-Graduação em Saúde Coletiva da Universidade Federal de Goiás – Brasil. Pós-Doutora em Saúde Coletiva pela Faculdade de Ciências Médicas da Universidade Estadual de Campinas – Brasil.; II Docente de Enfermagem na Escola Superior de Enfermagem de Lisboa – Portugal. Investigadora no Centro de Investigação, Inovação e Desenvolvimento em Enfermagem de Lisboa, Lisboa, Portugal.; III Docente de Enfermagem na Escola Superior de Enfermagem de Lisboa – Portugal. Doutora em Enfermagem. Investigadora no Centro de Investigação, Inovação e Desenvolvimento em Enfermagem de Lisboa, Lisboa, e no Centro de Inovação em Tecnologias e Cuidados de Saúde, Leiria, Portugal.

## INTRODUÇÃO

Em geral, os estudos de implementação na saúde e na enfermagem, em particular, enfrentam desafios que refletem a complexidade dos contextos clínicos e organizacionais, bem como a interação entre os diferentes atores envolvidos na produção, disseminação e aplicação do conhecimento^(1)^. A complexidade dos sistemas de saúde e das relações interpessoais e institucionais, reconhecida pela própria Organização Mundial da Saúde^(2)^, impede que a tomada de decisão em saúde seja, de forma consistente, informada pelas melhores evidências disponíveis, considerando simultaneamente as preferências e valores das pessoas e das suas famílias, além da perceção social, justiça, viabilidade de implementação, acesso equitativo, sustentabilidade e aceitação dos envolvidos^(1-3)^.

A solução para essa problemática complexa passa pelo envolvimento efetivo dos destinatários dos cuidados de saúde em todas as fases do processo de translação do conhecimento, desde o planeamento e desenvolvimento dos projetos até a implementação e manutenção do uso do conhecimento, visando gerar impacto societal^(1^ ^,^ ^3)^. Assim, a investigação deve não só produzir novos resultados, mas também promover uma compreensão profunda das necessidades dos contextos e das pessoas, identificando barreiras e facilitadores à transferência do conhecimento^(3)^.

Diante do exposto, a investigação qualitativa se revela fundamental para compreender as perceções, motivações e barreiras vividas por todos os atores envolvidos ao longo das diferentes fases do processo de translação do conhecimento^(1)^. Essa abordagem permite adaptar as estratégias às realidades concretas dos serviços de saúde, favorecendo uma prática mais reflexiva, contextualizada e participativa.

Em contextos de elevada complexidade, os estudos de implementação que integram uma abordagem qualitativa assumem um papel essencial na compreensão de como as intervenções planeadas — sejam políticas públicas, programas de saúde ou práticas pedagógicas — se materializam no terreno, permitindo também avaliar a sua eficácia. Contrariando a visão positivista prevalente, segundo a qual a eficácia se mede apenas por indicadores numéricos, a investigação qualitativa procura compreender o “porquê” e o “como” das ações e experiências humanas. Desse modo, essa abordagem se torna indispensável, pois ilumina significados, perceções e relações humanas que sustentam qualquer processo de mudança^(1,3)^, contribuindo para que a práxis em saúde seja orientada por conhecimento atualizado e contextualizado.

Tal perspetiva amplia a compreensão sobre como as evidências são transformadas em ação e sustentadas nos diferentes contextos de cuidado. Nos estudos de implementação, este olhar qualitativo possibilita compreender por que uma intervenção bem desenhada não produz o impacto esperado ou como uma prática aparentemente simples é reinterpretada ou apropriada por profissionais e comunidades. Dessa forma, a investigação qualitativa oferece um espelho das dinâmicas sociais, culturais e organizacionais que moldam a realidade da implementação^(3)^.

A adoção de abordagens naturalistas, interpretativistas e holísticas constitui, portanto, um imperativo. Estas promovem o aprofundamento da análise, enriquecem o conhecimento e a inovação metodológica, sustentando uma tomada de decisão ética e interdisciplinar. Tais características têm, provavelmente, contribuído para o crescente destaque dos estudos de métodos mistos, que procuram integrar o melhor dos dois paradigmas — quantitativo e qualitativo —, em uma visão mais abrangente e complementar.

Nesse contexto, a vertente qualitativa assume um papel central, não apenas pela sua capacidade de aprofundar a compreensão dos fenômenos, mas também pelo seu potencial de transformação nas práticas e nas relações em saúde. A investigação qualitativa tem ainda um efeito transformador nos estudos de implementação, uma vez que dá voz e espaço aos diferentes participantes — pessoas cuidadas e suas famílias, além de profissionais, gestores e decisores políticos —, promovendo uma ciência mais democrática, inclusiva e participativa. Mais do que produzir conhecimento sobre a implementação, a investigação qualitativa contribui para aperfeiçoar os próprios processos, ao permitir que os intervenientes reflitam criticamente sobre as suas práticas. Essa dimensão reflexiva transforma a investigação em um espaço de aprendizagem e de construção de sentido.

Em suma, designar a investigação qualitativa como a “alma” dos estudos de implementação não constitui apenas uma metáfora poética, mas um reconhecimento do seu papel essencial na compreensão das dimensões humanas da mudança. As políticas e programas só adquirem vida quando são interpretados, adaptados e vividos pelas pessoas. É a escuta atenta, o olhar sensível e a análise profunda que revelam o que os números não mostram: os significados que sustentam as ações. Assim, a investigação qualitativa não é apenas um método, mas uma forma de ver e compreender o mundo — uma ciência do humano que ilumina a verdadeira alma da implementação.

# Translation: Investigación cualitativa: el corazón de los estudios de implementación

**DOI:** 10.1590/0034-7167.20267902es

**Authors:** Ellen Synthia Fernandes de Oliveira, Maria Helena Presado, Cristina Lavareda Baixinho

**Affiliations:** I Profesora del Programa de Postgrado en Salud Pública de la Universidade Federal de Goiás – Brasil. Postdoctorado en Salud Pública por la Facultad de Ciencias Médicas de la Universidade Estadual de Campinas – Brasil.; II Profesora de Enfermería en la Escola Superior de Enfermagem de Lisboa – Portugal. Investigadora del Centro de Investigação, Inovação e Desenvolvimento em Enfermagem de Lisboa, Lisboa, Portugal.; III Profesora de Enfermería en la Escola Superior de Enfermagem de Lisboa – Portugal. Doutora em Enfermagem. Investigadora del Centro de Investigação, Inovação e Desenvolvimento em Enfermagem de Lisboa, Lisboa, y del Centro de Inovação em Tecnologias e Cuidados de Saúde, Leiria, Portugal.

## INTRODUÇÃO

En general, los estudios de implementación en salud, y en enfermería en particular, enfrentan desafíos que reflejan la complejidad de los contextos clínicos y organizacionales, así como la interacción entre los diferentes actores involucrados en la producción, difusión y aplicación del conocimiento^(1)^. La complejidad de los sistemas de salud y de las relaciones interpersonales e institucionales, reconocida por la propia Organización Mundial de la Salud^(2)^, impide que la toma de decisiones en salud esté informada consistentemente por la mejor evidencia disponible, considerando simultáneamente las preferencias y valores de los individuos y sus familias, así como la percepción social, la equidad, la viabilidad de implementación, el acceso equitativo, la sostenibilidad y la aceptación de los involucrados^(1-3)^.

La solución a este complejo problema radica en la participación efectiva de los destinatarios de la atención sanitaria en todas las fases del proceso de traducción del conocimiento, desde la planificación y el desarrollo de proyectos hasta la implementación y el mantenimiento del uso del conocimiento, con el objetivo de generar impacto social^(1,3)^. Por tanto, la investigación no sólo debe producir nuevos resultados, sino también promover una comprensión profunda de las necesidades de los contextos y de las personas, identificando barreras y facilitadores para la transferencia de conocimiento^(3)^.

A la luz de lo anterior, la investigación cualitativa resulta fundamental para comprender las percepciones, motivaciones y barreras que experimentan todos los actores involucrados a lo largo de las diferentes fases del proceso de traducción del conocimiento^(1)^. Este enfoque permite adaptar las estrategias a las realidades específicas de los servicios de salud, promoviendo una práctica más reflexiva, contextualizada y participativa.

En contextos de alta complejidad, los estudios de implementación que incorporan un enfoque cualitativo juegan un papel esencial para comprender cómo las intervenciones planificadas —ya sean políticas públicas, programas de salud o prácticas educativas— se materializan en la práctica, permitiendo además evaluar su efectividad. Contrariamente a la visión positivista predominante, según la cual la eficacia se mide únicamente mediante indicadores numéricos, la investigación cualitativa busca comprender el “por qué” y el “cómo” de las acciones y experiencias humanas. De esta forma, este enfoque se torna indispensable, pues ilumina significados, percepciones y relaciones humanas que fundamentan cualquier proceso de cambio^(1,3)^, contribuyendo para que la práctica en salud sea guiada por conocimientos actualizados y contextualizados.

Esta perspectiva amplía la comprensión de cómo la evidencia se transforma en acción y se sustenta en diferentes contextos de atención. En los estudios de implementación, este enfoque cualitativo permite comprender por qué una intervención bien diseñada no produce el impacto esperado o cómo una práctica aparentemente simple es reinterpretada o apropiada por profesionales y comunidades. De esta manera, la investigación cualitativa ofrece un reflejo de las dinámicas sociales, culturales y organizacionales que configuran la realidad de la implementación^(3)^.

Por lo tanto, es imperativo adoptar enfoques naturalistas, interpretativos y holísticos. Estos enfoques promueven el análisis profundo, enriquecen el conocimiento y la innovación metodológica, y apoyan la toma de decisiones ética e interdisciplinaria. Estas características probablemente han contribuido al creciente auge de los estudios de métodos mixtos, que buscan integrar lo mejor de ambos paradigmas —cuantitativo y cualitativo— en una visión más integral y complementaria.

En este contexto, el enfoque cualitativo asume un papel central, no sólo por su capacidad de profundizar la comprensión de los fenómenos, sino también por su potencial para transformar las prácticas y las relaciones en la atención de salud. La investigación cualitativa también tiene un efecto transformador en los estudios de implementación, ya que da voz y espacio a diferentes participantes (aquellos que reciben cuidados y sus familias, así como profesionales, gerentes y formuladores de políticas), promoviendo una ciencia más democrática, inclusiva y participativa. Más allá de la simple generación de conocimiento sobre la implementación, la investigación cualitativa contribuye a la mejora de los propios procesos, al permitir a los actores reflexionar críticamente sobre sus prácticas. Esta dimensión reflexiva transforma la investigación en un espacio de aprendizaje y construcción de significado.

En resumen, designar la investigación cualitativa como el “alma” de los estudios de implementación no es una mera metáfora poética, sino un reconocimiento de su papel esencial en la comprensión de las dimensiones humanas del cambio. Las políticas y los programas solo cobran vida cuando son interpretados, adaptados y vividos por las personas. La escucha atenta, la mirada sensible y el análisis profundo revelan lo que los números no muestran: los significados que sustentan las acciones. Por lo tanto, la investigación cualitativa no es solo un método, sino una forma de ver y comprender el mundo — una ciencia de lo humano que ilumina la verdadera esencia de la implementación.
